# Tetraspanins, More than Markers of Extracellular Vesicles in Reproduction

**DOI:** 10.3390/ijms21207568

**Published:** 2020-10-14

**Authors:** Jana Jankovičová, Petra Sečová, Katarína Michalková, Jana Antalíková

**Affiliations:** Laboratory of Reproductive Physiology, Institute of Animal Biochemistry and Genetics, Centre of Biosciences, Slovak Academy of Sciences, 84005 Bratislava, Slovakia; jana.jankovicova@savba.sk (J.J.); petra.secova@savba.sk (P.S.); katarina.michalkova@savba.sk (K.M.)

**Keywords:** sperm, oocyte, embryo, oviductosomes, uterosomes, epididymosomes, prostasomes, fertilization

## Abstract

The participation of extracellular vesicles in many cellular processes, including reproduction, is unquestionable. Although currently, the tetraspanin proteins found in extracellular vesicles are mostly applied as markers, increasing evidence points to their role in extracellular vesicle biogenesis, cargo selection, cell targeting, and cell uptake under both physiological and pathological conditions. In this review, we bring other insight into the involvement of tetraspanin proteins in extracellular vesicle physiology in mammalian reproduction. We provide knowledge regarding the involvement of extracellular vesicle tetraspanins in these processes in somatic cells. Furthermore, we discuss the future direction towards an understanding of their functions in the tissues and fluids of the mammalian reproductive system in gamete maturation, fertilization, and embryo development; their involvement in mutual cell contact and communication in their complexity.

## 1. Introduction

**Figure 1:**
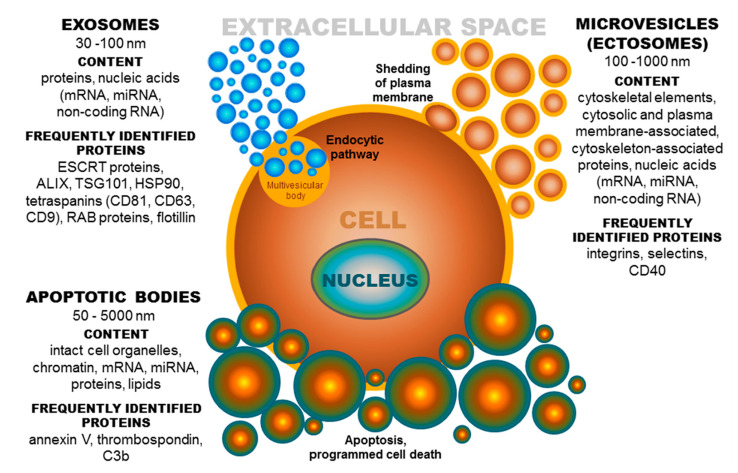
Extracellular vesicles: Their origin, size, and cargo. ESCRT-endosomal sorting complex required for transport; ALIX-protein regulating cellular mechanisms, including endocytic membrane trafficking and cell adhesion; TSG101-tumor susceptibility gene 101 protein; HSP-heat shock protein, CD-cluster of differentiation; RAB-proteins included in regulation of endocytosis and secretory processes, C3b-complement component.

Extracellular vesicles (EVs) are small membrane-derived particles released from cells into the extracellular space. The EV environment ensures the protection of cargo from enzymatic degradation during transit through the extracellular space [1,2,3]. EVs are released by most cell types under both normal and pathological conditions [4], and their presence has been observed in many body fluids [5,6,7]. It is known that the formation of extracellular vesicles is a precisely regulated process [8]. Based on published data, the term EVs denotes a population of different groups of vesicles classified according to their biogenesis and release pathway, evidently overlapping in some cases. Individual membrane vesicle categories differ, not only in origin, size, and morphology, but also in content [4] (Figure 1). The currently known data regarding the molecular cargo of extracellular vesicles (lipids, RNAs, and proteins) are summarized in the ExoCarta database [9,10,11] and in Vesiclepedia, a compendium for EVs [12,13]. Based on their diameter, EVs can be classified into several groups, namely, ectosomes, or shedding microvesicles (MVs) (100–1000 nm) [14,15,16]; exosomes (EXs) (30–100 nm) [15]; apoptotic bodies (ABs) (50–5000 nm) [17,18]; and other EV subsets, as reported by Shah et al. [19]. These groups of extracellular vesicles also differ in their origin [8]. Exosomes have an endocytic origin and are released from multivesicular endosomes. The biogenesis of EXs begins with the internalization of molecules via endocytosis [20]. Subsequently, endocytosed molecules are either recycled to the plasma membrane or trafficked to multivesicular bodies [4]. Multivesicular bodies fuse with the plasma membrane, leading to the release of intraluminal vesicles as exosomes into the extracellular microenvironment [21]. Whereas, MVs are formed by blebbing of the plasma membrane and subsequent fission of the membrane blebs [4,22], ABs are shed from the membrane of cells during the process of programmed cell death or apoptosis [4,18].

While it is generally accepted that the main function of EVs is the mediation of intercellular communication [23,24,25], they are also involved in cell homeostasis, coagulation, and waste management [22]. Although cells typically release several major EV populations defined by biophysical properties and biological functions, the heterogeneity among them is obvious and likely underlies the specific role of EV subpopulations in individual cellular processes [26]. EVs are effective intercellular transporters of proteins, lipids, and nucleic acids. The protein cargo of extracellular vesicles includes proteins participating in cell adhesion (integrins, ICAM (intracellular adhesion molecule)), intracellular trafficking (GTPases, RAB (proteins included in regulation of endocytosis and secretory processes, annexins), and signal transduction (protein kinases, G proteins, β catenin) [27]. EVs are usually enriched in tetraspanin proteins (mainly CD9, CD63, and CD81) and other proteins, such as ALIX (protein regulating cellular mechanisms), TSG101 (tumor susceptibility gene 101 protein), MHC1 (major histocompatibility complex 1), and HSP90 (heat shock protein 90) [28,29]. Regarding lipids, EVs are characterized by the presence of phosphatidylserine, cholesterol, sphingomyelins, and ceramides [30,31], which participate not only in intercellular signaling, but also ensure structural stability [32]. EVs may also carry nucleic acids (genomic DNA, mitochondrial DNA, mRNA, miRNA, and long non-coding RNA) [33]. Overall, EVs can alter the physiological and pathological function of recipient and parent cells through the transfer of proteins, lipids, and RNA [34]. Exosome uptake (and likely uptake of the other types of EVs) can cause activation, differentiation, or dedifferentiation of target cells depending on the delivered cargo [35]. Notably, it was shown that exosomal mRNA transferred to recipient cells can be translated and that miRNA may regulate gene expression in recipient cells [33]. Beside the somatic (body) systems, many tissues and cells of the reproductive tract release EVs, which are believed to participate in various steps of the reproduction process (reviewed in Reference [36]). The participation of tetraspanin family proteins, the most prevalent proteins in EVs [37,38,39], which are routinely used only as markers (mostly for exosomes) in mammalian fertilization, has been demonstrated [40,41,42]. In this review, we focus on current knowledge regarding EV tetraspanins, regarding reproduction, and their demonstrated or predicted function.

## 2. Tetraspanin Family Proteins

**Figure 2:**
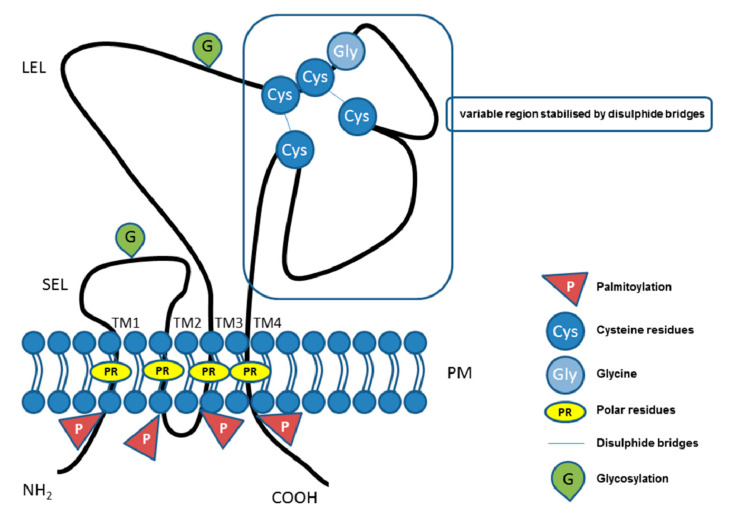
Illustrative schema of tetraspanin structure. Tetraspanin proteins traverse the plasma membrane (PM) four times, thus, defining the four transmembrane domains (TM1, TM2, TM3, TM4) with conserved polar residues (PRs) in their structure. In the extracellular space, the small (SEL) and large (LEL) extracellular loops can be recognized. The LEL contains a highly conserved CCG motif and possibly an additional two, four, five, six, or eight conserved cysteine residues (Cys). Between the cysteine residues, two disulfide bridges that enable folding of the LEL can be formed. Tetraspanins are post-translationally modified by glycosylation (G) in the large or small extracellular domain and palmitoylation (P) at the intracellular cysteine residues. Short *N*-terminal and *C*-terminal tails are oriented intracellularly.

In mammals, the tetraspanin protein family includes more than 30 members (Table 1), and tetraspanins have been found on the plasma membrane or in endosomal or lysosomal compartments of almost all cell types [43]. The tetraspanin family includes distinct proteins characterized by their common specific molecular structure (Figure 2), represented by four transmembrane domains containing conserved polar residues; a small extracellular loop (SEL) and large extracellular loop (LEL) with 4, 6, 7 or 8 conserved cysteine residues; and short cytoplasmic tails [44,45,46,47,48]. Polar residues stabilize the tertiary structure [49]. Tetraspanins are usually post-translationally modified by glycosylation in extracellular domains and palmitoylation at intracellular cysteines [43]. They also contain a tyrosine-based sorting motif for intracellular compartment targeting that may mediate internalization via associated proteins [50]. Tetraspanins are generally accepted to have an important role as organizers of each other and of distinct transmembrane and cytosolic proteins (integrins, members of the immunoglobulin superfamily, proteases) into a multimolecular membrane network called the tetraspanin web [48,51,52,53,54]. Association with proteins and lipids results in the organization of specific microdomains located in membranes that are different from lipid rafts, so-called tetraspanin-enriched microdomains (TEMs). Within the tetraspanin web, several levels of tetraspanin protein associations have been described. Through specific, lateral association, tetraspanins can create primary complexes with other non-tetraspanin proteins. Tetraspanins also possess a strong predisposition to associate with each other, which likely contributes to the assembly of a secondary interaction network in which non-tetraspanin proteins are associated via palmitoylated tetraspanins acting as linker proteins. Moreover, tetraspanins can associate with lipids (e.g., cholesterol), creating larger complexes [43]. It was shown that the constant region of the LEL is responsible for tetraspanin dimerization, while the variable region is responsible for the association with non-tetraspanin partners [49]. In general, tetraspanins are implicated in many cellular processes, such as cell adhesion [55], regulation of cell motility, and/or morphology, fusion, signaling, and other functions [52,56,57,58,59]. Increasing evidence points to their pivotal role in the pathogenesis of viral, bacterial, parasitic, and fungal infections (reviewed in References [60,61,62]) and in cancer (reviewed in References [63,64]). Moreover, it was shown that tetraspanins are important, if not essential, in mammalian fertilization [40,41,42].

## 3. Tetraspanins and Other Proteins in Extracellular Vesicles (EV Tetraspanin Network?)

Because of the high content of tetraspanins, such as CD9, CD81, CD63, and CD82, in EXs, they are most often used as specific exosome markers [65,66,67]. However, since tetraspanin expression has also been detected on cell surfaces, they may be a part of plasma membrane-derived vesicles, such as microvesicles, directly budded from the membrane. In addition to tetraspanins (e.g., CD9, CD81, CD82, CD63, CD151, tetraspanin-6, and tetraspanin-8), EVs also possess other proteins. Willms et al. [26] reported that while MVs were found to be selectively enriched in cytoskeletal elements, cytoskeleton-associated proteins (e.g., actin, actinin, dynamin, myosins, tubulin, and VDAC1/2) and septins (along with their associated binding partners), EXs were enriched in integrins (α_2_, α_6_, β_1_, and β_4_) and other proteins (syntenin, TSG101, and ALIX). In the same study, after summarizing the extensive proteomic analysis of EXs, the authors stated that exosomes released by distinct cell types and even from the same cells can largely differ both quantitively and qualitatively in their specific tetraspanin profile. These data suggest that the unique composition of individual tetraspanins in EVs may be responsible for alterations in cellular responses.

Although several protocols and methods are used for EV isolation (differential ultracentrifugation, density gradient flotation, size exclusion chromatography, ultrafiltration, asymmetrical-flow field-flow fractionation, microfluidic isolation, flow cytometry, and immunocapture (reviewed in Reference [26])), most methods result in a fraction enriched by some population of EVs, but ‘contaminated’ by other extracellular vesicle populations, and thus, currently, there is a lack of reliable assays for isolation and characterization approaches that address EV heterogeneity. With the aim of improving research in the field of extracellular vesicles, Lozano-Andrés et al. [68] modified synthetic nanovesicles with recombinant antigenic regions of tetraspanin proteins enriched in EVs (CD9, CD81, and CD63) to provide reference material for the detection of EVs. It is important to note that tetraspanins are involved in protein biogenesis, exosome production, and vesicular cargo sorting, and thus, they significantly affect the function of vesicles.

## 4. Role of EV Tetraspanins in Somatic Cells

### 4.1. Role of Tetraspanins in EV Formation

Exosomes can be formed through ESCRT (endosomal sorting complex required for transport)-dependent or ESCRT-independent pathways (reviewed in References [29,69,70,71]). Studies by van Niel et al. [71] and Chairoungdua et al. [72] indicated that tetraspanins may be crucial players in ESCRT-independent pathways of exosome biogenesis and secretion. It is well known that tetraspanins can modulate membranes by affecting their curvature [73,74,75,76], which directly predetermines their participation in EV formation. Considering the reversed cone-like molecular shape of CD9, Umeda et al. [74] suggested that clustering of tetraspanin molecules could modulate membrane curvature to enable exosome budding and facilitate subsequent interaction of the C-terminal region of tetraspanins (cytoplasmic tail) with cytoskeletal actin through ezrin, radixin and moesin proteins [77], which may be involved in EV fission from the parent cell membrane.

### 4.2. Role of Tetraspanins in EV Cargo Selection, Targeting, and Uptake

Although the processes of targeting and uptake (internalization of EVs by recipient cells) are currently not fully understood, it is evident that molecules involved in tetraspanin-enriched microdomains of EVs and TEMs of recipient cells, especially tetraspanins, integrins, and other adhesion proteins, play a key role in the process of binding, fusion, and targeting of extracellular vesicles and in selective uptake of EVs by recipient cells [26].

As shown in exosomes, the internalization of membrane molecules into endosome compartments is related to the rearrangement of TEMs [78]. Based on previous findings, Rana et al. [38] hypothesized that regulation of the protein assembly of exosomes, and potentially, the recruitment of microRNA is ensured by tetraspanins. In 2012, Rana et al. [39] reported for the first time that selective exosome uptake by cells and tissues is critically dependent on the tetraspanin web composition. Therefore, it seems that TEMs are involved not only in molecular internalization and recycling, but that tetraspanin proteins (at least CD9, CD81, CD82, CD63, and tetraspanin-8) also regulate the sorting of proteins and possibly RNA to EVs (reviewed in Reference [79]). A quantitative proteomic analysis conducted by Perez-Hernandez et al. [80] showed that the TEM interaction network corresponds to 45% of the exosomal proteome. Consequently, the diminishment of the tetraspanin profile of extravesicular TEMs can result in a decrease in the concentration of some of their associated partners. In general, common components of TEMs within the cell membrane are tetraspanins, integrins, and other adhesion receptors and transmembrane receptor proteins [52,57,81,82]. Therefore, changes in the expression of specific tetraspanins may modulate selective targeting and uptake of EVs, thereby affecting the cellular response. Willms et al. [26] suggested the utilization of the tetraspanin (and integrin) profile as a distinguishing criterion for individual EV subpopulations. The involvement of tetraspanins in vesicular cargo selection was also reported in neuroblastoma cells, where EVs targeted the cells depending on the presence of CD63 tetraspanin or amyloid precursor protein [31]. The exosomal tetraspanins CD9 and CD81, together with integrin α_v_β_3_, were shown to be involved in the targeting and uptake of exosomes by dendritic cells, and a complex of tetraspanin-8 with integrin subunit CD49d (α_4_) determines the selective targeting and uptake of tumor-derived exosomes by endothelial cells [83]. In a very recent study, Umeda et al. [74] revealed the cone-like molecular shape of CD9 (based on the crystal structure of CD9 and cryo-electron microscopy of human CD9) and proposed two potential roles of tetraspanins in exosome function. Tetraspanin clustering may directly affect membrane curvature and thereby enable exosome budding, or alternatively, tetraspanins can control vesicular cargo sorting through association with other partner proteins via EWI family proteins [77,84], thereby establishing a complex functional molecular network [74].

To deliver proteins or nucleic acids, EVs undergo direct fusion with the target cell membrane or the endosomal compartment membrane after endocytic uptake [85,86,87,88]. The uptake of EVs can occur through several endocytic pathways, including clathrin-dependent endocytosis and clathrin-independent pathways, such as caveolin-mediated uptake, macropinocytosis, phagocytosis, and lipid raft-mediated internalization. The same authors stated that the different uptake mechanism of individual EV populations likely depends on the proteins and glycoproteins on the surface of the vesicle and the target cell (reviewed in Reference [87]). Based on the fact that tetraspanins are involved in many cellular processes, including vesicular and cellular fusion [52,89], they are ‘predestined’ to participate in EV binding and uptake by cells (reviewed in References [90,91,92,93]). In vitro and in vivo experiments have suggested that exosomes diffuse throughout the whole body, with selective enrichment in different cells/organs depending on exosome-tetraspanin complexes and cell ligands [39]. These findings were supported by experiments on somatic cells, where a reduction in EV uptake by target/recipient cells was observed after treatment with antibodies against tetraspanin-8, CD9, CD81, and CD151 [94,95,96]. It should be noted that in some cases, a cellular response, such as activation/inhibition of signaling pathways, may be induced by EVs without their internalization, as has been illustrated in several studies [97,98,99,100,101,102]. It is generally believed that tetraspanins act as regulators of the adhesive activity of several adhesion molecules, including integrins [103,104,105,106,107,108,109], and thus, they may not only control adhesion, but may also affect signaling pathways. Therefore, specific tetraspanin and integrin profiles of EVs could ensure the specific role of individual EV subpopulations.

### 4.3. Role of Tetraspanins in Immunostimulatory and Immunosuppressive Properties of EV Subpopulations in Cancer

In recent years, great effort has been devoted to the elucidation of EV function in cancer biology (reviewed in References [110,111]). Numerous EVs secreted by cancer cells transport their molecular content to various target cells, including endothelial, epithelial, and immune cells, and serve as regulators of intercellular communication of cancer cells [110,112]. Increasing experimental data indicate a crucial role of EVs in cancer progression. On the other hand, several studies have suggested that non-tumoral cells may suppress cancer initiation and progression via EVs (reviewed in Reference [110]). Extracellular vesicles can participate in remodeling of the tumor microenvironment, which affects endothelial cells, epithelial cells, stromal cells, fibroblasts, and macrophages [113,114,115]. Findings regarding tetraspanins and integrins are also related to their role in organotropism, the non-random distribution of metastases in an organ-specific pattern [39,113,116]. Hoshino et al. [116] suggested that integrins in EVs mediate not only their adhesion to recipient cells, but also trigger signaling and inflammatory responses in recipient cells, thereby leading organs to become permissive for metastatic cell growth. As reported by Yue et al. [113], the communication between tumor-derived EVs and the matrix is also significantly affected by the interaction of tetraspanins (CD151 and tetraspanin-8) with integrins, and proteases. In addition, EVs can mediate hematopoietic and stromal cell activation, including (lymph) angiogenesis, and stimulate epithelial-mesenchymal transition in neighboring non-metastatic tumor cells [113].

### 4.4. Role of EV Tetraspanins in Antigen Presentation

Most immune system cells (likely all), including antigen-presenting cells (APCs), secrete extracellular vesicles, and depending on the type and status of the parent cell, EVs may modulate the immune response in various ways (initiation, expansion, maintenance, or silencing) [117]. Professional antigen-presenting cells mainly include dendritic cells, macrophages, and B cells. It was shown that EVs released by APCs can induce an adaptive immune response by the distribution of MHC-peptide complexes and presentation of these antigens to specific T cells ([21,118,119], as reviewed in Reference [79]). As known molecular organizers, tetraspanins present on the surface of immune cells have been shown to play an important role in the adaptive immune response via involvement in antigen presentation and formation of molecular complexes in immunological synapse assembly [89,120,121,122,123,124,125]. The interaction of the APC tetraspanins CD9, CD37, CD53, CD81, and CD82 with MHC molecules, as well as engagement of MHC-peptide complexes in TEMs, has led to the consideration of their role in the formation of MHC-II multimers and enhanced antigen presentation [122,126,127,128,129]. According to Kropshofer et al. [122], the organization of MHC-II complexes in TEMs could determine the composition of the immunological synapse, and subsequently, the quality of the T helper cell response. Andreu and Yáñez-Mó [79] suggested that in addition to regulation of the expression and sorting of MHC to EVs, tetraspanins may be responsible for the degree of MHC complex clustering in EV membranes sufficient to induce an immune response. Tetraspanins can also participate in the regulation of different steps in the immune response through their ability to alter the function of their associated partners (reviewed in Reference [117]).

**Figure 3:**
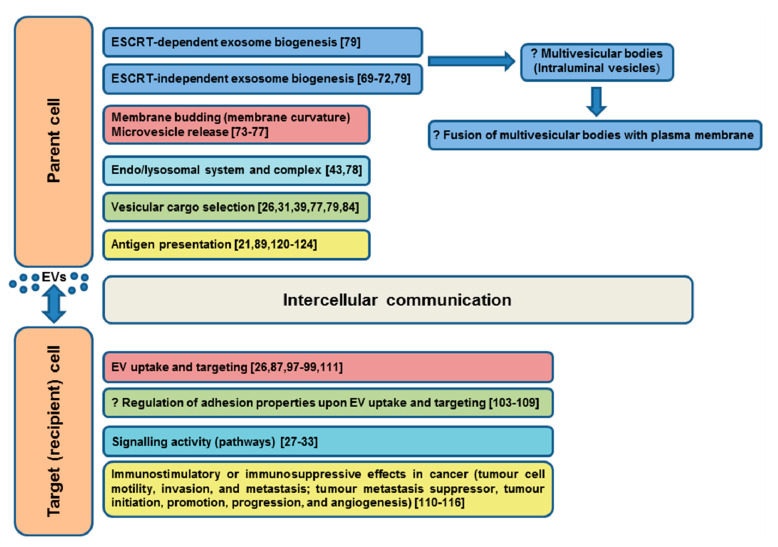
Evidenced/predicted involvement of tetraspanins in EV functions in somatic cells. EVs-extracellular vesicles, ESCRT-endosomal sorting complex required for transport; ?-predicted role.

The evidenced or predicted involvements of tetraspanins in EV functions in somatic cells are summarized in Figure 3.

## 5. Tetraspanins in EVs of the Female and Male Reproductive System

### 5.1. Female Reproductive System

In most animals, developing oocytes arrested in primordial ovarian follicles in meiotic prophase I are surrounded by granulosa cells and follicular fluid. From the moment of female reproductive maturity, the oocytes begin to form zona pellucida, and granulosa cells start proliferation and cumulus formation. After the resumption of meiosis, the first polar body is extruded, and the oocyte is arrested in metaphase II. The process within the follicle (called folliculogenesis) is mediated by paracrine factors from theca cells, mural and cumulus granulosa cells and the oocyte itself [130,131], and after ovulation (the process after follicle rupture when the egg is released into the oviduct), there is communication between the cumulus-oocyte complex and the oviductal epithelium. Moreover, differential secretion of oviductal proteins reflects the presence of an oocyte or spermatozoon [132]. To understand the physiological, as well as pathological events during mammalian gamete maturation, fertilization, and embryo development, it is essential to clarify the interactions of the individual components (hormones, growth factors, enzymes, and many other proteins) with gametes or the embryo upon their journey through the female reproductive tract. It has been suggested that one of the vehicles of bioactive material are EVs, but little is known about their function and molecular content, particularly regarding the role of tetraspanins, which are typically only referred to as markers of EVs.

#### 5.1.1. EVs in Follicular Fluid

Oocyte growth and development within the follicle is completely dependent on the nourishing capacity of the granulosa cells, and communication between follicular cells and oocytes occurs via paracrine and gap-junctional signaling [133]. The highly specialized cumulus cells (CCs) have a membranous extension and transzonal projections (TZPs), which penetrate the zona pellucida (ZP) and join to the oocyte plasma membrane, forming gap junctions at the ends of TZPs and between cumulus cells. These connections are essential for molecular transfer between the oocyte and CCs and among individual cumulus cells [134]. Currently, it is known that this communication is bidirectional [134,135], and the possibility of molecular transport mediated by extracellular vesicles has been discussed in previous studies [136,137].

The presence of EVs (CD63-positive) in ovarian follicular fluid (FF) was first identified in horses by da Silveira et al. [138]. The authors documented the transfer of bioactive material through the uptake of microvesicles and exosomes from the follicular fluid by surrounding granulosa cells. Their later findings indicated a possible role of exosomes from FF in the regulation of follicle maturation [139]. CD63-positive EV-mediated cell-cell communication within the follicle was also confirmed in cattle [140] and in pigs [141]. Later, the uptake of EVs (CD63-positive) by bovine granulosa and cumulus cells was documented, and EVs were identified in cumulus cells and in transzonal projections [142]. CD63- and CD81-positive exosomes have also been isolated from human FF [143,144]. The impact of EVs on ovarian function was documented for the first time by Hung et al. [145] in cattle. The authors found that highly CD81-enriched EVs derived from small but not large bovine follicles, likely released by granulosa cells, were able to upregulate cumulus gene expression in vitro, and thus, to stimulate cumulus expansion. Based on different levels of CD81 within EVs isolated from small, medium, and large follicles, Navakanitworakul et al. [146] suggested that changes in EV biogenesis or uptake occurred during follicle development.

Interestingly, a positive effect of EVs (CD63-positive) isolated from follicular fluid on correct bovine embryo development has been observed [142].

#### 5.1.2. Oocyte EVs

The presence of CD9-containing exosomes, such as vesicles in the perivitelline space (PVS, the space between the oolema and ZP) of mature (MII) mouse oocytes was reported by Miyado et al. [147], and the existence of a CD81-rich layer in the ZP and a CD9-rich layer in the PVS of mouse oocytes was documented by Ohnami et al. [148]. The authors stated that the CD9 layer is predominantly produced in oocytes, while the CD81 layer is predominantly produced in cumulus cells and localized to the ZP [148]. In cattle, extracellular vesicles were detected outside the TZP within the perivitelline space of oocytes, and communication of cumulus cells and oocytes via these cell-secreted vesicles was suggested [136]; later, CD9-positive structures resembling transzonal projections were observed in MII bovine oocytes [149].

Thus, it can be assumed that EVs assist in the initial stages of follicle and oocyte development and maturation.

#### 5.1.3. Oviductal EVs

Successful maturation of a developmentally competent oocyte is followed by its release from the ovary into the oviduct, a part of the female reproductive tract that connects the ovary with the uterus and provides the opportunity for transport and contact between female and male gametes, followed by successful fertilization and early embryo development. After ovulation, the cumulus-oocyte complex (COC) passes through the fallopian tube and ampulla to the ampullary-isthmic junction, the place where contact with sperm occurs [150]; thus, the oviductal fluid is present not only during the time of ovulation, but also at the moment of sperm-egg interaction and following fertilization events. Oviductal EVs (oEVs), or ‘oviductosomes’, as they were named by Al-Dossary et al. [151], have recently been identified as potential mediators of the gamete/embryo interaction within the oviduct. They have been described in mice [151,152], cows [153,154], women [155], hens [156], bitches [157], and turtles [158]. Nakano et al. [159] reported that CD81-expressing exosomes from oviductal mesenchymal cells likely modulate the ciliogenesis of Müllerian epithelial cells in mice. CD9-positive oEVs were detected in mouse oviducts [152] and in bovine oviductal fluid secreted by oviductal epithelial cells in vivo and by these cells cultivated in vitro [154]. Interestingly, the presence of CD9-positive oEVs remained unchanged across the different stages of the cow estrous cycle [160], although proteomic analysis showed different protein profiles of in vivo and in vitro secreted EVs [154] and that their different cargo is under hormonal regulation [160]. Moreover, dissimilar concentrations and sizes of oEVs obtained from different parts of the oviduct (ampulla and isthmus) were described [161]. The isthmus is a part of the mammalian oviduct serving as a sperm reservoir (reviewed in Reference [150]), where sperm undergo their final maturation process, called capacitation, involving a series of many biochemical and physiological changes essential for the acquirement of fertilization competence (reviewed in Reference [162]). The importance of oEVs in this process was documented by Al-Dossary et al. [151], who showed not only the presence, but also the role of CD9-positive oEVs in mouse sperm acquisition of PMCA4a protein, a member of a family of Ca^2+^ efflux pumps essential for the hyperactivated motility and fertility of sperm. The same authors later determined the mechanism of this cargo delivery. They proposed that sperm-oEV fusion is mediated by receptor-ligand interactions of α_ν_β_3_ and α_5_β_1_ integrins in sperm and in oEVs, where CD9 acts as a scaffold for these adhesion molecules [163]. The presence of oEVs carrying and delivering PMCA4 was also documented later in humans [155].

Before the sperm reach the oviduct, they must traverse the uterus, where they meet extracellular vesicles called ‘uterosomes’ [164]. In addition to the previously mentioned PMCA4 protein from oviductosomes, the sperm can gain the same molecule together with GPI-linked SPAM1 (Sperm adhesion molecule 1) [151,164] from uterosomes (CD9-positive) during this transport.

It is known that the oviductal mucosa protects sperm against aging damage during storage within the mammalian isthmus. Moreover, the positive effect of vesicles prepared from the apical cell membranes of the oviductal epithelium on sperm viability was documented twenty years ago in rabbits [165], horses [166], and humans [167]. In avian species, the unique ability of sperm to stay in oviductal tubular crypts for a prolonged period is also known [168], but the mechanism by which the sperm are protected in sperm storage tubules (SSTs) of the hen oviduct is still not fully understood. Huang et al. [156] suggested that the high CD63 protein expression level in the SSTs may be physiologically related to the sperm storage function of the oviduct. The authors detected CD63 protein in the apical region and cytoplasm of SST cells from the control group and artificially inseminated group of hens; they observed a decreasing level of CD63 in SST cells containing sperm. The reduction in CD63 protein was not observed in SST cells without sperm, suggesting secretion of CD63-containing exosomes by SSTs into the lumen in response to resident sperm and their probable role during the storage of sperm in the oviduct [156]. Higher secretion of CD63-positive vesicles by ciliated and gland cells of oviductal epithelium at the time of sperm storage was also reported in the Chinese soft-shelled turtle *Pelodiscus sinensis* [158].

#### 5.1.4. EVs of Fertilized Oocytes and Early Embryos

In addition to MII oocytes, the presence of extracellular vesicles has also been documented in the moment of fertilization in mice. Transfer of CD9-positive oocyte fragments to fertilizing spermatozoa was observed by Barraud-Lange et al. [169]. Later, Miyado et al. [147] reported contact of sperm with CD9-positive vesicles released from the oocyte PM into the PVS of oocytes and their ability to facilitate sperm-egg fusion. Similar findings were also described in hamsters. The authors suggested that the structures within the inner part of the ZP may support this transfer via the formation of a complex with CD9-positive vesicles. Interestingly, the suggestion of Miyado et al. [147] that the transfer of CD9-containing vesicles from wild-type oocytes could rescue the fertility of *Cd9*-deficient mice was later questioned by Gupta et al. [170] and Barraud-Lange et al. [171]. The release of CD9-positive vesicles in mice was also observed immediately after gamete fusion, when CD9 leaves the oocyte PM together with other associated proteins, and thus, likely participates in the prevention of polyspermy [172]. CD9-positive vesicles in the ZP after fertilization were documented in human zygotes [173] and in the PVS of bovine and porcine embryos [149]. Additionally, CD81-positive vesicle clusters were observed in the PVS of bovine [174] and porcine [149] embryos.

#### 5.1.5. EVs in Later Embryo Development and Implantation

During the early post-fertilization stage, the embryo undergoes several developmental changes, such as the first cell division, activation of the embryonic genome, compaction of the morula, and formation of the blastocyst. Under physiological conditions, these processes begin in the oviduct, where the embryo spends from two to six days, depending on the species [175]. EVs produced on both sides play an important role in mutual oviduct-embryo communication, which ensures proper embryo development and subsequent implantation [176].

EVs isolated from embryonic and maternal sources have been described by several authors. In addition to the aforementioned secretion of oviductosomes [151], EV secretion by endometrium cells of the uterus has been documented in mice [164], humans [177,178], and sheep [179]. Moreover, EVs produced by embryos in vitro or in vivo have been reported in cattle [180,181] and sheep [179]; however, their study in terms of structure, protein content, and role in embryo development is difficult. At least in cattle, differences in the secretion and protein composition of EVs under in vivo and in vitro conditions have been described [154]. Nevertheless, in bovine embryos, it was shown that internalized oEVs (containing CD9) during in vitro culture improved the potential of embryos to reach the blastocyst stage and survive under in vitro conditions [153,154,161]. Similarly, in mice, EVs containing CD9 derived from donor oviductal fluid improved embryo transfer efficiency [182].

Despite the fact that a bidirectional embryo-maternal communication has been suggested in cattle [154,183,184], in humans, only the uptake of embryonal EVs (eEVs) by endometrial epithelial cells, but not by oviductal cells has been shown [185]. This could be due to the problem in distinguishing oEVs from eEVs obtained from in vivo sources (oviductal fluid and maternal plasma [186]). Moreover, the population of EVs secreted by the embryo is species-specific, and their production also differs depending on embryo production technology and embryo developmental stage [173,181,187,188].

As regards embryonal EVs in terms of tetraspanin presence, CD9-positive eEVs secreted by porcine embryos produced in vitro and their role as a tool for communication between embryos (and also within their microenvironment) in vitro was reported for the first time by Saadeldin et al. [189]. The presence of CD9- and CD63-positive EVs secreted from day 7 to 9 by in vitro cultured bovine blastocysts (both by in vitro fertilization and parthenogenetic activation derived blastocysts) was subsequently reported by Mellisho et al. [181]. The embryo-embryo cross-talk was also confirmed in bovine embryos cultured in groups [190], where vesicles (CD9- and CD63-positive) isolated from embryo-conditioned culture medium were internalized by embryonic cells and had a positive effect on blastocyst development and lowering apoptotic cell ratios. Moreover, the essentiality of EVs (CD9-positive) secreted by bovine embryos cloned using somatic cell nuclear transfer technology for their further development was also documented by Qu et al. [191]. In addition, CD81- and CD63-positive EVs from human blastocoel fluid [192] and secretion of EVs strongly positive for CD9 and CD81 and weakly positive for CD63 and CD82 by in vitro cultured bovine embryos were recently reported [193].

Under physiological conditions, the embryo leaves the oviduct and passes to the uterus, or it is inserted into the uterus after in vitro fertilization. Successful implantation occurs in the microenvironment of the uterine cavity and is influenced by several factors. After entering the uterus, the blastocyst undergoes hatching and final preparation for implantation, during which its apposition, adhesion, and invasion through the endometrial epithelium occur. An important role in the process of attachment to the endometrium is played by the trophectoderma (layer of trophoblasts), which later forms the maternal component of the placenta [178]. Production of EVs during contact of the embryo with the endometrial epithelial cells of the uterus and uterine fluids [177] was confirmed, and the importance of mutual communication through those EVs for proper implantation, placentation, further embryonic development, and thus, a normal pregnancy was reported (reviewed in Reference [175]).

In humans, CD9-, CD63- and CD81-positive EVs have been observed in uterine fluid [177,194]. As a source of EVs in the uterine cavity, endometrial epithelial cells with CD9 and CD63 on their apical surface have been detected. The intensity of CD63 staining reached a maximum in the middle secretory phase of the menstrual cycle, the time of endometrial receptivity for implantation, while CD9 expression did not change [177]. The role of CD9 as a regulator of α_3_, α_6_, and β_1_ integrins in association with endometrial function in embryo implantation was reported earlier by Park et al. [195]. In endometrial cell cultures, CD81 and CD151 tetraspanins were detected along with variable levels of several integrins, which are known as tetraspanin partners. While β_1_, α_3_, and α_6_ laminin-binding integrins were highly expressed, α_2_ integrins showed intermediate expression, ανβ_3_ integrins showed low expression levels, and α_1_, α_5_, β_2_, and α_4_ integrins showed very low or undetectable expression levels [196]. Moreover, the authors described the CD98 receptor, predominantly associated with CD9 within TEMs, as a putative determinant of endometrial receptivity during embryo implantation in humans. Overexpression of these two proteins significantly enhanced mouse blastocyst adhesion. Additional tetraspanin-14 was identified in EVs released from human endometrial epithelial cells (luminal and glandular) by Greening et al. [178]. These EVs were taken up by trophoblast cells and enhanced their adhesive capacity during implantation. The authors also considered the important role of EVs in cell-cell communication in the microenvironment of the uterus before implantation and placentation and hypothesized that EVs from a single endometrial epithelial cell could affect surrounding endometrial cells, and thus, prepare them for implantation.

In mice, the presence of CD9 in the endometrium, especially in cells surrounding early implantation sites, was documented by Wynne et al. [197]; however, according to these authors, maternal CD9 expression is not essential for successful embryo implantation or maintenance of pregnancy. According to Liu et al. [198], CD9 expressed in the endometrium acts rather as a suppressor at the invasion but not the adhesion stage of embryo implantation. On the other hand, it was suggested a supportive effect of CD9-positive EVs derived from outgrowth embryo on mouse embryonic developmental competence in vitro and implantation potential in vivo [187]. A higher blastocyst formation rate of embryos co-cultured with exosomes (CD9-, CD63-positive) and a reduction in the apoptotic rate of these embryos in pregnant mice [199] has also been documented.

Nakamura et al. [200] and later, Kusama et al. [201] focused on studying EVs in the uterine fluid in cattle. They also confirmed that the source of EVs in this fluid is not only the endometrium, but also the fetus and that these EVs are necessary for interactions between the embryo and the endometrium, particularly in the implantation process. In addition to CD63, a large number of other exosomal proteins have been identified, including ezrin and moesin [200], the connection of which with other tetraspanins (predominantly CD9) in complex protein network and vesicle formation has been recently documented [74]. Mutual maternal-embryo communication through CD63-positive EVs isolated from uterine fluid has also been observed in sheep [202].

Similarly, in pigs, the release of CD63-positive EVs by the endometrium and chorioallantoic membrane/placenta and by porcine trophectoderm cells has been documented [203,204]. Moreover, communication between the EVs secreted by the trophectoderm and internalized by endothelial cells of the endometrium and vice-versa has been hypothesized in this species, taking into account the unique epitheliochorial (non-invasive) placentation [204].

The mammalian placenta is a temporary organ consisting of fetal and maternal tissues, connecting the developing fetus to the uterine wall, with the interface mediating nutrient uptake, thermo-regulation, waste elimination, and gas exchange. It serves as a source of hormones and as an immunological barrier. The importance of EVs in the communication of placental cells with maternal tissues has been documented in the later stages of pregnancy in humans. In exosomes (CD63-positive) produced by the placenta, several bioactive molecules (RNA, DNA, proteins, and peptides) have been detected, and their release from chorionic villous trophoblasts into maternal circulation has been confirmed [205]. The importance of placental exosomes as a communication tool contributing to placentation and development of maternal-fetal vascular exchange has been suggested. In addition, the possibility of utilizing the exosome profile in the diagnosis of placental dysfunction was proposed by Salomon et al. [206]. An increased number of CD63-positive placental exosomes in maternal plasma during the first trimester of normal pregnancy was reported by Sarker et al. [207], and their pathophysiological relevance in preeclampsia (PE) was suggested by Redman et al. [208] and Baig et al. [209]. Pillay et al. [210] considered exosomes (CD63-positive) as a useful tool for prognosis/diagnosis of PE, even allowing clinicians to distinguish two subtypes of preeclampsia (early onset and late-onset PE), and thus, helpful in clinical decisions about the timing of childbirth in PE. In addition, Salomon et al. [211] documented the relationship between placental EVs and gestational diabetes mellitus (GDM) and suggested that the differential release of exosomes from the placenta and other maternal tissues compared to healthy patients affects the endothelial cell response (cytokine release), and thus, the pro-inflammatory state of GDM. CD63-positive exosomes secreted by human placenta carrying immunomodulators that modify the maternal immune system have also been reported [212]. Furthermore, Zhang et al. [213] suggested that EVs (with CD9, CD81, and CD63 markers) can also be used for early diagnoses of fetal genetic diseases, such as fetal trisomies, chromosomal abnormalities, and de novo mutations in fetuses, thanks to the similarity of the fetal DNA in EVs from maternal plasma and cell-free DNA in maternal blood.

Burkova et al. [214] identified 11 major proteins (including tetraspanin CD81) and 27 different peptides and small proteins (molecular size 2–10 kDa) in human placental exosomes. The authors hypothesized the importance of exosome peptides for protective pathways in the mother and in the development of embryos and newborns.

### 5.2. Male Reproductive Tract

#### 5.2.1. Epididymal EVs-Epididymosomes

Testicular spermatozoa, although fully differentiated, are unable to fertilize. They are released to the epididymis, which is a highly ordered and segmented tubular organ of the male genital tract divided into three regions with distinctive functions: caput, corpus, and cauda [215]. The heterogeneous cell types lining its lumen, the distribution, structure, and function of which differ along the length of the duct, ensure the processes contributing to the main role of the epididymis, which is physiological maturation of the spermatozoa, leading to motility and the ability to fertilize the oocyte (reviewed in Reference [216]). While early and late sperm maturational events occur in the caput and corpus, the cauda region primarily serves as a reservoir for functionally mature spermatozoa. Each of these segments possesses distinctive gene expression profiles within the epithelium, which results in the segment-specific secretion of proteins into the luminal fluid that directly or indirectly affect sperm maturation (reviewed in Reference [217]). Sperm epididymal maturation is associated with morphological, biochemical, and physiological changes, such as the incorporating new molecules derived from the epididymal epithelium and post-translational modifications of endogenous proteins synthesized during spermiogenesis in the testis. These processes result in a sperm progressive movement, migration of the cytoplasmic droplet, changes in the sperm proteome, and changes in the sperm surface (reviewed in Reference [218]), including an increase in total negative surface charge, modifications to lectin-binding properties, changes in membrane lipid composition, remodeling of raft membrane microdomains and of other plasma membrane structures, modifications of surface glycoproteins, and surface antigen relocalization (reviewed in Reference [219]). The mutual communication of different epididymal epithelial cell types (principal, narrow, clear, and basal cells) generates a luminal microenvironment appropriate for sperm maturation and storage in the epididymis. An important role within these processes is attributed to EVs called epididymosomes, which are released by principal cells into the lumen of the epididymal duct (reviewed in Reference [218]). Epididymosomes have been observed in the epididymal fluid of hamsters [220], rats [221], sheep [222], mice [223], monkeys [224], cattle [225], and humans [224]. Epididymosomes are defined as small membranous vesicles (25–300 nm in diameter) that contain different proteins, lipids, and non-coding RNAs (reviewed in Reference [218]). Animal and human studies have provided much evidence for the contribution of EVs to sperm maturation. However, to date, there is limited evidence for the roles of specific types of EVs (reviewed in Reference [36]).

Epididymosomes collected from different segments of the epididymis are highly heterogeneous in size and content, which may explain differences in protein transfer. Epididymosomal proteins can either be incorporated into the sperm plasma membrane or into intracellular structures (reviewed in Reference [218]). It was reported that compartmentalization of proteins in epididymosomes is critical for the coordination of protein transfer to specific sperm compartments or structures [226]. Nevertheless, the exact mechanism by which epididymosomes transfer proteins to sperm remains elusive [227]. Knowing that tetraspanin proteins ensure compartmentalization of proteins in membranes of other somatic cells, it is likely that they participate in epididymal sperm maturation, at least in this manner. Thimon et al. [228] analyzed the protein composition of human epididymosomes isolated from epididymal fluid collected during the vasovasostomy procedure and identified 146 proteins, including the tetraspanins CD9, CD81, CD63, and tetraspanin-1. Moreover, the protein composition of epididymosomes differed from that of prostasomes. Recently, a study by Nixon et al. [229] presented a comparative proteomic analysis of epididymosomes isolated from different segments of the mouse epididymis, and a total of 1640 proteins, including the tetraspanins CD9, CD63, CD81, CD82, CD151, tetraspanin-6, tetraspanin-8, tetraspanin-9, and tetraspanin-14, were identified and quantified. They also demonstrated the uptake of protein cargo of mouse epididymosomes, primarily into the head of caput sperm.

Based on the presence of the tetraspanin CD9, two distinct populations of epididymosomes were characterized in the bovine epididymal fluid. The CD9-positive population of vesicles fused with spermatozoa in complex with P25b and GliPriL1 (Glioma pathogenesis-related 1-like protein 1), both known to be involved in the sperm-egg interaction, and with MIF (Macrophage migration inhibitory factor) and AKR1B1 (Aldo-keto reductase family 1 member B1), proteins involved in sperm motility. These vesicles preferentially bound to or fused with live sperm, in contrast to the other vesicle population, which was enriched in ELSPBP1 (epididymal sperm binding protein 1) and had a high affinity for dead spermatozoa. Although both EV populations bound to the same sperm regions (acrosome and midpiece) with the same kinetics [230], CD9-positive vesicles fused with sperm via a tetraspanin complex and transferred proteins important for sperm maturation, while the other vesicles likely protected live spermatozoa against ROS generated by dying spermatozoa through the scavenger activity of BLVRA (Biliverdin Reductase A), a partner of ELSPBP1 [231]. Some epididymal proteins are anchored to spermatozoa by glycosylphosphatidylinositol (GPI) residues [232]. Typically, a GPI anchor is attached to the nascent protein in the endoplasmic reticulum [233], but there is experimental evidence that some newly acquired proteins are GPI anchored to spermatozoa during epididymal transit. One of them is P25b in cattle [225] or P26h in hamsters, which has been mentioned previously [234]. Another one is a CD52-like molecule (bovine maturation-associated sperm membrane antigen), detected by Michalková et al. [235]. Based on the assumption of Lefèvre et al. [236], who suggested an association between tetraspanins and GPI proteins, and the findings of de Gassart et al. [237], who reported clustering of GPI-anchored proteins and membrane-associated ligands into microdomains on the surface of EVs (exosomes), one could expect tetraspanin ‘assistance’ in the transport of GPI proteins on the epididymal sperm membrane via EVs.

#### 5.2.2. EVs in Semen

Mammalian semen (seminal fluid) released during ejaculation consists of spermatozoa and seminal plasma, the mix secreted by the epididymis, rete testis, and accessory sex organs/glands (prostate gland, seminal vesicles, and bulbourethral glands also known as Cowper’s glands). The composition of seminal fluid differs not only among species, but also between males of one species and even between ejaculates of the same male. Currently, it is clear that in addition to the major components, such as peptides, proteins, enzymes, hormones, cytokines, lipids, sugars, and ions (reviewed in Reference [238]), EVs interacting with spermatozoa are undoubtedly also a part of seminal plasma (reviewed in Reference [239]). While Rolland et al. [240] reported that human seminal plasma contains 2545 unique proteins, Yang et al. [239] identified only a total of 1474 proteins, of which 58.6% (864) were overlapping with the human seminal plasma exosomal proteome, and suggested that their biological role was likely related to metabolism, energy pathways, cell growth and maintenance, and transport. In human ejaculate, eleven subcategories of EVs were observed, and 59% were single vesicles, and 41% consisted of multifaceted formations [241]. Seminal plasma provides not only a suitable medium to convey sperm to the female genital tract and ensures sperm survival and function, but also regulates the female immune system (reviewed in Reference [242]). Although the exact physiologic role of EVs in seminal plasma has not yet been fully elucidated, it is believed that EVs are involved in many of these processes (reviewed in Reference [243]). EVs in seminal plasma can be distinguished by origin as epididymosomes, prostasomes, or vesicles of seminal plasma, which represents a mix of EVs with an undefined origin.

#### 5.2.3. Prostasomes

Prostasomes were first identified in human seminal fluid as organelles in prostatic secretion [244,245], and therefore, were named ‘prostasomes’ [246]. They were described as intracellular microvesicles (50–500 nm in diameter) inside another larger vesicle, a so-called storage vesicle, equivalent to multivesicular bodies of late endosomal origin. Prostasomes are produced by the endosome-containing epithelial cells lining the acinar ducts within the tissue of the prostate gland. The fusion between the membrane surrounding the storage vesicle and the plasma membrane of the prostate acinar cell (exocytosis) enables the release of prostasomes into prostatic fluid [246,247]. Prostasomes can be involved in fertilization in several ways. They can participate in the promotion of sperm forward motility and hyperactivation through the regulation of Ca^2+^ homeostasis. Prostasomes can protect spermatozoa from reactive oxygen species and against the female immune system through complement inhibitory activity, they have antibacterial properties and can regulate sperm capacitation through cholesterol transfer, and they have been implicated in the acrosome reaction (reviewed in Reference [248]).

Although there are several studies on tetraspanins regarding epididymosomes, prostasomes, and other populations of EVs in seminal plasma, tetraspanins are mostly used as markers of EVs and not as an object of interest.

Aalberts et al. [249] found the tetraspanin CD9 in human prostasomes and later CD9, specifically associated with 50–150 nm vesicles [250]. Du et al. [251] observed a positive effect of CD9- and CD63-positive EVs (exosomes) isolated from boar seminal plasma on several parameters of sperm ‘quality’. Sperm showed prolonged effective motility time, improved plasma membrane integrity and antioxidant capacity, and inhibited premature capacitation. However, it should be noted that it is not known which molecules are responsible for these effects. Brzozowski et al. [252] demonstrated that alteration of CD9 and CD151 on prostate cells changed the proteome of derived EVs and that these EVs can subsequently enhance the migration and invasiveness of a non-tumorigenic population of prostate cells. The presence of exosomes (CD9-, CD81-, CD63-positive) in all fractions of boar seminal plasma was confirmed by Alvarez-Rodriguez et al. [242].

Barranco et al. [253] documented different tetraspanin expression profiles on exosomes and microvesicles of boar semen, observing that the proportion of EVs expressing CD63 and CD9 was higher in microvesicles than in exosomes, in contrast to CD81, which indicated distinct cargo, binding, and roles of EV subpopulations. These findings emphasized the need to directly focus on the role of EV tetraspanins in a broader sense rather than merely as EV markers.

Interestingly, Alvarez-Rodriguez et al. [254] were not able to detect CD9 (or CD44) in chicken seminal plasma, and they observed no or rare extracellular vesicles, suggesting that interaction of avian seminal fluid with the female genital tract occurs in a manner distinct from that in mammals.

## 6. Conclusions

**Figure 4:**
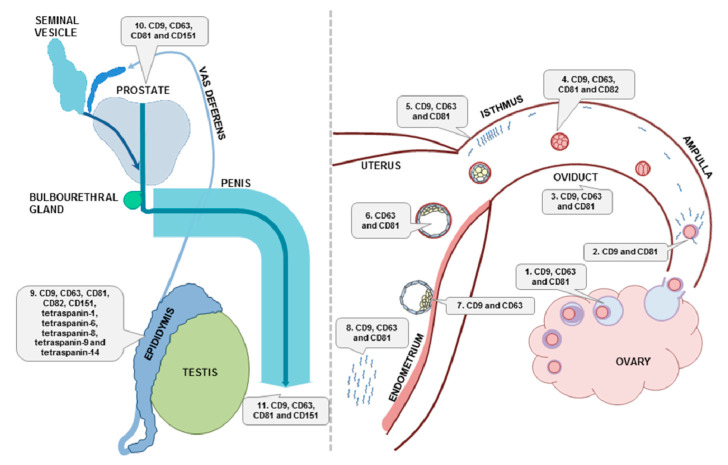
Schematic presentation of tetraspanins detected in extracellular vesicles within mammalian male and female reproductive systems. 1. CD9, CD63, and CD81 on EVs in follicular fluid during oocyte maturation; 2. CD9 and CD81 in EVs during fertilization; 3. CD9, CD63, and CD81 in EVs of oviductal fluid; 4. CD9-, CD63-, CD81-, and CD82-positive EVs produced by the early embryo; 5. CD9, CD63, and CD81 in EVs in the sperm reservoir in the isthmus; 6. CD63 and CD81 in EVs of blastocoel fluid; 7. CD9 and CD63 in EVs during embryo implantation; 8. CD9-, CD63-, and CD81-positive EVs in uterine fluid; 9. CD9, CD63, CD81, CD82, CD151, tetraspanin-1, tetraspanin-6, tetraspanin-8, tetraspanin-9, and tetraspanin-14 in EVs of epididymal fluid; 10. CD9, CD63, CD81, and CD151 in EVs related to prostate secretion; 11. CD9, CD63, CD81, and CD151 in EVs of semen.

Extensive research in the field of extracellular vesicles has shown their participation via their molecular cargo in many cellular functions in mammals. Although currently, tetraspanin proteins are applied mostly as markers of extracellular vesicles, consistent with the recommendation of the International Society for Extracellular Vesicles [255], increasing evidence points to their pivotal role in EV biogenesis, cargo selection, cell targeting, and uptake, which underlies their effect on distinct cellular processes under both physiological and pathological conditions. Notably, tetraspanins have been shown to be involved in sperm-egg interaction. Moreover, mammalian cells of both the male and female reproductive systems produce different EV populations, and their role in the processes of gamete maturation, fertilization, and embryo development has been suggested. These findings emphasize the need to consider the significance of tetraspanins regarding the message they transport. The data regarding the presence of tetraspanin-positive EVs during reproduction are summarized and illustrated in Figure 4.

Understanding the enigmatic role of EV tetraspanins is challenging, due to their complexity. Their individual abundance differs among species, individuals, organs, and anatomical regions and is dependent on sexual maturity and the presence of gametes or an embryo (e.g., oocyte, sperm, or early embryo in the oviduct; sperm or embryo in the uterus). Moreover, their study within reproductive organs is complicated by the occurrence of vesicle populations from various sources at the same time in the same place (e.g., EVs from ejaculate and EVs from the uterus after ejaculation or EVs of the uterus and embryo after fertilization). In addition, the possible differences in EV content produced by in vitro and in vivo cultivated oocytes or embryos should be taken into consideration.

Researchers working in the field of EV tetraspanins in reproductive physiology face many difficulties related to their function in multimolecular complexes. Their composition likely differs depending on the processes in which they are involved.

In sum, the constantly updated knowledge regarding extracellular vesicles has provided substantial findings that markedly change the point of view on physiological, as well as pathological processes, and the contribution of EV tetraspanins should not be omitted.

## Figures and Tables

**Table 1:** List of human tetraspanin superfamily members. Data were acquired from the UniProt database (UniProt Consortium. (2019). UniProt: A worldwide hub of protein knowledge. Nucleic acids research, 47(D1), D506-D515).

Protein Name	Alternative Name (s)	Gene Name
Tetraspanin-1	Tetraspan NET-1 (neuroepithelial cell-transforming gene 1 protein), Tetraspanin TM4-C	*TSPAN1*
Tetraspanin-2	Tetraspan NET-3	*TSPAN2*
Tetraspanin-3	Tetraspanin TM4-A, Transmembrane 4 superfamily member 8	*TSPAN3*
Tetraspanin-4	Novel antigen 2 (NAG-2), Transmembrane 4 superfamily member 7	*TSPAN4*
Tetraspanin-5	Tetraspan NET-4, Transmembrane 4 superfamily member 9	*TSPAN5*
Tetraspanin-6	A15 homologue, Putative NF-kappa-B-activating protein 321, T245 protein, Tetraspanin TM4-D, Transmembrane 4 superfamily member 6	*TSPAN6*
Tetraspanin-7	Cell surface glycoprotein A15, Membrane component chromosome X surface marker 1, T-cell acute lymphoblastic leukemia-associated antigen 1 (TALLA-1), Transmembrane 4 superfamily member 2, CD231	*TSPAN7*
Tetraspanin-8	Transmembrane 4 superfamily member 3, Tumor-associated antigen CO-029	*TSPAN8*
Tetraspanin-9	Tetraspan NET-5	*TSPAN9*
Tetraspanin-10	OCULOSPANIN	*TSPAN10*
Tetraspanin-11	-	*TSPAN11*
Tetraspanin-12	Tetraspan NET-2, Transmembrane 4 superfamily member 12	*TSPAN12*
Tetraspanin-13	Tetraspan NET-6, Transmembrane 4 superfamily member 13	*TSPAN13*
Tetraspanin-14	DC-TM4F2, Transmembrane 4 superfamily member 14	*TSPAN14*
Tetraspanin-15	Tetraspan NET-7, Transmembrane 4 superfamily member 15	*TSPAN15*
Tetraspanin-16	Tetraspanin TM4-B, Transmembrane 4 superfamily member 16	*TSPAN16*
Tetraspanin-17	F-box only protein 23, Tetraspan protein SB134, Transmembrane 4 superfamily member 17	*TSPAN17*
Tetraspanin-18	-	*TSPAN18*
Putative tetraspanin-19	-	*TSPAN19*
Uroplakin-1b (UP1b)	Tetraspanin-20 (Tspan-20)	*UPK1B*
Uroplakin-1a (UP1a, UPKa)	Tetraspanin-21 (Tspan-21)	*UPK1A*
Peripherin-2	Retinal degeneration slow protein, Tetraspanin-22 (Tspan-22)	*PRPH2*
Rod outer segment membrane protein 1 (ROSP1)	Tetraspanin-23 (Tspan-23)	*ROM1*
CD151 antigen	CD151, GP27, Membrane glycoprotein SFA-1, Platelet-endothelial tetraspan antigen 3 (PETA-3), Tetraspanin-24 (Tspan-24)	*CD151*
Leucocyte surface antigen CD53	CD53, Cell surface glycoprotein CD53, Tetraspanin-25 (Tspan-25)	*CD53*
Leucocyte antigen CD37	CD37, Tetraspanin-26 (Tspan-26)	*CD37*
CD82 antigen	CD82, C33 antigen, IA4, Inducible membrane protein R2, Metastasis suppressor Kangai-1, Suppressor of tumorigenicity 6 protein, Tetraspanin-27 (Tspan-27)	*CD82*
CD81 antigen	CD81, 26 kDa cell surface protein TAPA1, Tetraspanin-28 (Tspan-28), Target of the antiproliferative antibody 1	*CD81*
CD9 antigen	CD9, 5H9 antigen, Cell growth-inhibiting gene 2 protein, Leukocyte antigen MIC3, Motility-related protein (MRP-1), p24, Tetraspanin-29 (Tspan-29)	*CD9*
CD63 antigen	CD63, Granulophysin, Lysosomal-associated membrane protein 3 (LAMP-3), Melanoma-associated antigen ME491, OMA81H, Ocular melanoma-associated antigen, Tetraspanin-30 (Tspan-30)	*CD63*
Tetraspanin-31 (Tspan-31)	Sarcoma-amplified sequence	*TSPAN31*
Tetraspanin-32 (Tspan-32)	Protein Phemx	*TSPAN32*
Tetraspanin-33 (Tspan-33)	Penumbra (hPen), Proerythroblast new membrane	*TSPAN33*

## Abbreviations

ABs	Apoptotic bodies
AKR1B1	Aldo-keto reductase family 1 member B1
ALIX	ALG-2 interacting protein X (programmed cell death 6 interacting protein)
APCs	Antigen-presenting cells
BLVRA	Biliverdin reductase A
CCs	Cumulus cells
CD	Cluster of differentiation
COC	Cumulus-oocyte complex
eEVs	Embryonal extracellular vesicles
ELSPBP1	Epididymal sperm binding protein 1
ESCRT	Endosomal sorting complex required for transport
EVs	Extracellular vesicles
EWI	Proteins, members of immunoglobulin superfamily
EXs	Exosomes
FF	Follicular fluid
GDM	Gestational diabetes mellitus
GliPriL1	Glioma pathogenesis-related 1-like protein 1
GPI	Glycosylphosphatidylinositol
HSP	Heat shock proteins
ICAM	Intracellular adhesion molecule
LEL	Large extracellular loop
MHC	Major histocompatibility complex
MIF	Macrophage migration inhibitory factor
MII	Metaphase II
MVs	Microvesicles
oEVs	Oviductal extracellular vesicles
PE	Preeclampsia
PMCA4	Plasma membrane calcium ATPase 4
PVS	Perivitelline space
RAB	Proteins included in regulation of endocytosis and secretory processes
ROS	Reactive oxygen species
SEL	Small extracellular loop
SPAM	Sperm adhesion molecule
SSTs	Sperm storage tubules
TEMs	Tetraspanin-enriched microdomains
TSG101	Tumor susceptibility gene 101 protein
TZPs	Transzonal projections
VDAC1/2	Voltage-dependent anion-selective channel protein 1/2
ZP	Zona pellucida
